# Association of dietary carbohydrate and fiber ratio with postmenopausal bone mineral density and prevalence of osteoporosis: A cross-sectional study

**DOI:** 10.1371/journal.pone.0297332

**Published:** 2024-02-14

**Authors:** Lushuang Zhang, Liubiqi Zhao, Xinyu Xiao, Xiaobin Zhang, Li He, Qiang Zhang

**Affiliations:** 1 Department of Obstetrics and Gynecology, Chengdu Women’s and Children’s Central Hospital, School of Medicine, University of Electronic Science and Technology of China, Chengdu, China; 2 Department of Gynecology, Guangxi Guigang people’s Hospital, Guigang, China; University of Campania Luigi Vanvitelli: Universita degli Studi della Campania Luigi Vanvitelli, ITALY

## Abstract

**Background:**

This study aimed to investigate the associations of carbohydrate to dietary fiber ratio with bone mineral density (BMD) and the prevalence of osteoporosis in postmenopausal women.

**Methods:**

This cross-sectional study retrieved the data of 2829 postmenopausal women from the National Health and Nutrition Examination Survey (NHANES) database. Weighted univariable logistic regression models were used to investigate the correlations of carbohydrate, dietary fiber, or carbohydrate to fiber ratio with osteoporosis.

**Results:**

Higher dietary fiber intake was correlated with decreased odds ratio of osteoporosis [odds ratio(OR) = 0.96, 95% confidence interval (CI): 0.93 to 0.99]. The odds ratio of osteoporosis in postmenopausal women was elevated as the increase of carbohydrate to fiber ratio (OR = 1.80, 95%CI: 1.10 to 2.96). Carbohydrate to fiber ratio >17.09 was related to increased odds ratio of osteoporosis (OR = 1.63, 95%CI: 1.04 to 2.56). Compared to the carbohydrate to fiber ratio ≤11.59 group, carbohydrate to fiber ratio >17.09 was associated with decreased total femur BMD (β = -0.015, 95%CI: -0.028 to -0.001) and femur neck BMD (β = -0.020, 95%CI: -0.033 to -0.006) in postmenopausal women. The femur neck BMD in postmenopausal women was decreased with the increase of carbohydrate to fiber ratio (β = -0.015, 95%CI: -0.028 to -0.001).

**Conclusion:**

In postmenopausal women, a high carbohydrate/fiber ratio >17.09 is associated with an increased risk of osteoporosis and lower hip BMD and high fiber intake is associated with less osteoporosis and higher hip BMD.

## Introduction

Osteoporosis is a common systemic bone disease that causes loss of bone function, manifesting by decreased bone mineral density (BMD) in individuals [1]. Osteoporosis results in bone fragility and increased risk of fractures, which significantly impacts women’s quality of life and leads to physical, social, psychological, and financial consequences [2]. Postmenopausal osteoporosis is a prevalent disorder of bone metabolism that affects women following amenorrhea [3]. Postmenopausal osteoporosis is characterized by estrogen deficiency as well as persistent calcium loss with age, and the prevalence of osteoporosis was increased with age [4]. Previous evidence demonstrated that approximately 20% of females suffered from osteoporosis, with 10% of these individuals experiencing fractures in various locations [5]. To identify more reliable biomarkers associated with osteoporosis in postmenopausal women was of essential importance.

A diet containing adequate nutrients has been identified as a modifiable risk factor for osteoporosis, and there was growing interest in the effects of nutrients on bone health [6]. Nutritional supplements exerted a dominant role in skeletal health, both in achieving the highest BMD and in maintaining bone health, which are commonly used to prevent and treat osteoporosis, indicating a balanced diet and good nutritional program was important for osteoporosis [7]. Carbohydrate and fiber are two most important components of diet. Recent studies found that higher carbohydrate intake was associated with lower BMD [8]. As one of the components of carbohydrate, dietary fiber was identified to have potential beneficial to bone health [9, 10]. Higher dietary fiber intake was associated with higher calcaneal BMD [11]. The carbohydrate-to-fiber ratio was proposed as an indicator for assessing the quality of carbohydrate consumption, reflecting the equilibrium between refined grains, sugars, whole grains and bran contents [12]. Previous evidence showed that carbohydrate to dietary fiber ratio ≤10 was correlated to lower serum triglyceride triglyceride/high-density lipoprotein cholesterol, fasting insulin and insulin resistance levels [13]. There was an interaction between glucose metabolism, lipid metabolism and bone metabolism [14, 15]. Therefore, we suspected that carbohydrate to fiber ratio might be associated with bone metabolism in postmenopausal women.

This study aimed to validate the hypothesis that carbohydrate to dietary fiber ratio might be correlated with BMD and the prevalence of osteoporosis in postmenopausal women using the data from the National Health and Nutrition Examination Survey (NHANES). Subgroup analysis was performed in postmenopausal women in different body mass index (BMI) groups.

## Methods

### Study design and population

Our study was a cross-sectional study retrieved the data of 5309 postmenopausal women from the NHANES database between 2005 and 2018. The NHANES is a nationally representative cross-sectional survey of the non-institutionalized civilian resident United States (US) population, distinguished by its intricate sampling strategy [16]. Details of recruitment, procedures, population characteristics, and study design for NHANES are provided through the Centers for Disease Control and Prevention (https://www.cdc.gov/nchs/nhanes/index.htm). The data collection included a home interview and examinations in standardized physical mobile examination centers (MECs) every 2 years (https://wwwn.cdc.gov/nchs/nhanes/tutorials/module2.aspx). All participants completed home interviews, and the observers were from the same team, which decreased the observer variation. The study involved individuals with the assessment of femur neck and total femur BMD, and with complete information of carbohydrate and dietary fiber intake in the NHANES database. As BMD was assessed in 2005–2006, 2007–2008, 2009–2010, 2013–2014 and 2017–2018 in NHANES, and we used data from these cycles. The inclusion criteria of this study were (1) postmenopausal women, (2) participants with the assessment of femur neck and total femur BMD, (3) participants with complete information of carbohydrate and dietary fiber intake in the NHANES database. The exclusion criteria were (1) subjects without information of key covariates and (2) with unusually low or high total energy intake (<500 kcal/day or >5000 kcal/day) were excluded. Finally, 2829 postmenopausal women were included. The requirement of ethical approval for this was waived by the Institutional Review Board of Chengdu Women’s and Children’s Central Hospital, School of Medicine, University of Electronic Science and Technology of China, because the data was accessed from NHANES (a publicly available database). The need for written informed consent was waived by the Institutional Review Board of Chengdu Women’s and Children’s Central Hospital, School of Medicine, University of Electronic Science and Technology of China due to retrospective nature of the study.

### Potential covariates and definitions

General characteristics including age (years), race (Mexican American, other Hispanic, non-Hispanic White, non-Hispanic Black or other race-including multi-racial), education (less than 9th grade, 9-11th grade (Includes 12th grade with no diploma), high school graduate/general equivalent diploma (GED) or equivalent, some college or Associate of Arts (AA) degree or college graduate or above), marriage (married, widowed, divorced, separated, never married or living with partner), poverty-to-income ratio (<1.0, ≥1.0 or unknown), drinking (≤twice/week or >twice/week), smoking (yes or no), BMI (kg/m^2^), circumference (cm), cotinine (ng/mL), and physical activity [<450 metabolic equivalent of task (MET)·min/week, ≥450 MET·min/week or unknown], complications including hypertension (no/yes), diabetes (no/yes), previous fracture (no/yes), parental fracture (no/yes), and history of physician-diagnosed osteoporosis, eating habits including calcium values in laboratory (mg/dL), cotinine (ng/mL), 25-hydroxyvitamin D (25[OH]D) (nmol/L), phosphorus (mg/dL), serum calcium (mg), Vitamin D (mcg), and protein intake (gm), and drug use including glucocorticoid use (no/yes), alkaline-phosphatase (IU/L), or osteoporosis treatment, estrogens treatment, and bone active treatment including Nx drug (anticoagulants) and Kz drug (thyroid hormones) were potential covariates analyzed.

Physical activity was assessed by metabolic equivalent task (MET). MET × min = recommended MET × exercise time of corresponding activity (min) [17]. Hypertension was defined as people who have self-reported high blood pressure, systolic blood pressure ≥140 mmHg or diastolic blood pressure ≥90 mmHg, or who took blood pressure medications. Calcium was calculated based on the Calcium intake in Day 1 dietary recall data and supplements. Vitamin D intake was defined according to Vitamin D intake in Day 1 dietary recall data and supplements. BMI ≥25 kg/m^2^ was regarded as overweight.

### Main variables

Carbohydrate, dietary fiber and carbohydrate to fiber ratio were the main variables. Carbohydrate was calculated as carbohydrate/total energy (100gm/1000kcal), and grouped based on tertiles. Dietary fiber was calculated as dietary fiber/total energy (10gm/1000kcal) and grouped based on tertiles. Carbohydrate to fiber ratio = carbohydrate/dietary fiber and grouped based on tertiles.

### Assessment of dietary fiber and carbohydrate intake

The dietary intake data are utilized to estimate the types and quantities of foods and beverages (including all forms of water) consumed within the 24-hour period preceding the interview (from midnight to midnight), as well as to assess intakes of energy, nutrients, and other food components from those consumables. The dietary interview component, known as What We Eat in America (WWEIA), is conducted through a collaborative effort between the U.S. Department of Agriculture (USDA) and the U.S. Department of Health and Human Services (DHHS). Within this partnership, DHHS’ National Center for Health Statistics (NCHS) assumes responsibility for survey sample design and all aspects related to data collection, while USDA’s Food Surveys Research Group (FSRG) takes charge of dietary data collection methodology, maintenance of databases used for coding and processing data, as well as data review and processing [18]. All NHANES participants are eligible for two 24-hour dietary recall interviews. The first dietary recall interview is collected in-person in the Mobile Examination Center (MEC) and the second interview is collected by telephone 3 to 10 days later. Detailed information about each food/beverage item (including the description, amount of, and nutrient content) reported by each participant is included in the Individual Foods files (https://wwwn.cdc.gov/Nchs/Nhanes/2013-2014/DR1IFF_H.htm#DR1IFDCD). This method has been validated in large-scale studies and proven effective for accurately assessing nutrient intake among adults. For each participant, estimates of fiber and carbohydrate intake from each individual food or beverage item were calculated.

### Outcome variable

Osteoporosis was the outcome, which was determined by BMDs of total femur and femur neck according to DXXOFBMD and DXXNKBMD in DXXFEM, respectively. BMD was measured using dual-energy X-ray absorptiometry (DXA). A high level of quality control was maintained throughout the DXA data collection and scan analysis, including a rigorous phantom scanning schedule. Staff from the National Center for Health Statistics (NCHS) and the NHANES data collection contractor monitored technologist acquisition performance through in-person observations in the field. Retraining sessions were conducted with the technologists annually and as needed to reinforce correct techniques and appropriate protocol. In addition, technologist performance codes were recorded by the NHANES quality control center at the University of California, San Francisco (UCSF), Department of Radiology during review of participant scans. The codes documented when the technologist had deviated from acquisition procedures and where scan quality could have been improved. The performance codes were tracked for each technologist individually and a summary reported to NCHS on a quarterly basis. Additional feedback on technologist performance was provided by the UCSF when problems were noted during review of the scans. Constant communication was maintained throughout the year among the UCSF, the NCHS, and the data collection contractor regarding any issues that arose. Hologic service engineers performed all routine densitometer maintenance and repairs. Copies of all reports completed by the manufacturer’s service engineers were sent to the UCSF when the scanners were serviced or repaired so any changes in measurement as a result of the work could be assessed. Each participant and phantom scan was reviewed and analyzed by the UCSF using standard radiologic techniques and study-specific protocols developed for the NHANES. Expert review was conducted by the UCSF on 100% of analyzed participant scans to verify the accuracy and consistency of the results. Invalidity codes were applied by the UCSF to indicate the reasons femur and spine regions of interest (ROI) could not be analyzed accurately. The quality control phantoms were scanned according to a predetermined schedule. A number of data quality issues were addressed through the quality control program. The expert review procedures assured that scan analysis was accurate and consistent. (https://wwwn.cdc.gov/Nchs/Nhanes/2013-2014/DXXFEM_H.htm).

The T-score was calculated using BMD measurements at the femur neck and total femur (respondent’s BMD-reference group mean BMD)/reference group standard deviation (SD)). The reference group for the femoral neck comprised non-Hispanic White women aged 20–29 years from NHANES III [19]. Measured osteoporosis was defined as femur neck or total femur BMD T-score ≤ -2.5.

### Construction of the weighted logistic regression and linear regression model

The masked variance unit pseudo-stratum was sdmvstra, and the masked variance unit pseudo-primary sampling units was sdmvpsu. The confidence interval (CI) was applied for assess the reliability of an estimate. A set of weights WTDRD1 was used when an analysis uses the Day 1 dietary recall data. Weighted univariable logistic regression models were used to investigate the correlations of carbohydrate, dietary fiber, and carbohydrate to fiber ratio with osteoporosis. Variables with statistical difference (*P*<0.05) were regarded as confounding factors, and total energy was also adjusted ^19^. In Model 1, no variables were adjusted, in Model 2, age and race were adjusted, and in Model 3, age, race, education, marriage, poverty-to-income ratio, physical activity, hypertension, previous fracture, BMI, circumference, cotinine, protein intake, history of physician-diagnosed osteoporosis or osteoporosis treatment, estrogens treatment, and total energy were adjusted. Weighted univariable linear regression models were adopted to assess the associations of carbohydrate, dietary fiber and carbohydrate to fiber ratio with dietary fiber with total femur BMD or femur neck BMD. Variables with statistical difference (*P*<0.05) were regarded as confounding factors, and total energy was also adjusted. Model 1 adjusted for no variable, Model 2 adjusted for age and race, and Model 3 adjusted for age, race, education, marriage, poverty-to-income ratio, physical activity, hypertension, previous fracture, BMI, circumference, cotinine, protein intake, history of physician-diagnosed osteoporosis or osteoporosis treatment, estrogens treatment, and total energy.

### Statistical analysis

Kolmogorov-Smirnov was conducted to evaluate the normality of quantitative data. The normally distributed quantitative data were described as Mean (standard error) [Mean (SE)], and comparison between the two groups was compared by independent sample t test. Non-normally distributed data were described as median and quartiles [M (Q_1_, Q_3_)]. Categorical data were described as number and percentage of cases [n (%)], Chi-square test was used for comparison between groups, and rank sum test was used for rank data. The missing values of variables were presented in S1 Table. Due to the large proportion of missing values of poverty-to-income ratio and physical activity, the missing data was classified as unknown group. The remaining missing variables were manipulated based on the Random Forest chain equation multiple interpolation method, which were interpolated using the miceforest package in python. Sensitivity analysis revealed that there was no significant difference between the data before and after manipulation (S2 Table). With osteoporosis as an outcome, weighted univariable logistic regression models were used to investigate the correlations of carbohydrate, dietary fiber, or carbohydrate to fiber ratio with osteoporosis. With total femur BMD or femur neck BMD as outcomes, weighted linear regression models were adopted to assess the associations of carbohydrate, dietary fiber, or carbohydrate to fiber ratio with hip BMD respectively. Subgroup analysis was stratified by BMI. The interactions of BMI with carbohydrate, dietary fiber, or carbohydrate to fiber ratio on the odds ratio of osteoporosis in postmenopausal women were also evaluated. *P*<0.05 suggested the interaction was statistical different. Odds ratio (OR), β and 95% confidence interval (CI) were employed as effect size. All statistical tests were performed by a two-sided test with a test level of α = 0.05. Python 3.9 was used for missing value processing, and SAS 9.4 (SAS Institute Inc., Cary, NC, USA) was used for statistical analysis. *P*<0.05 was considered as statistically different.

## Results

### Comparisons of the characteristics of people with and without osteoporosis

**Fig 1:**
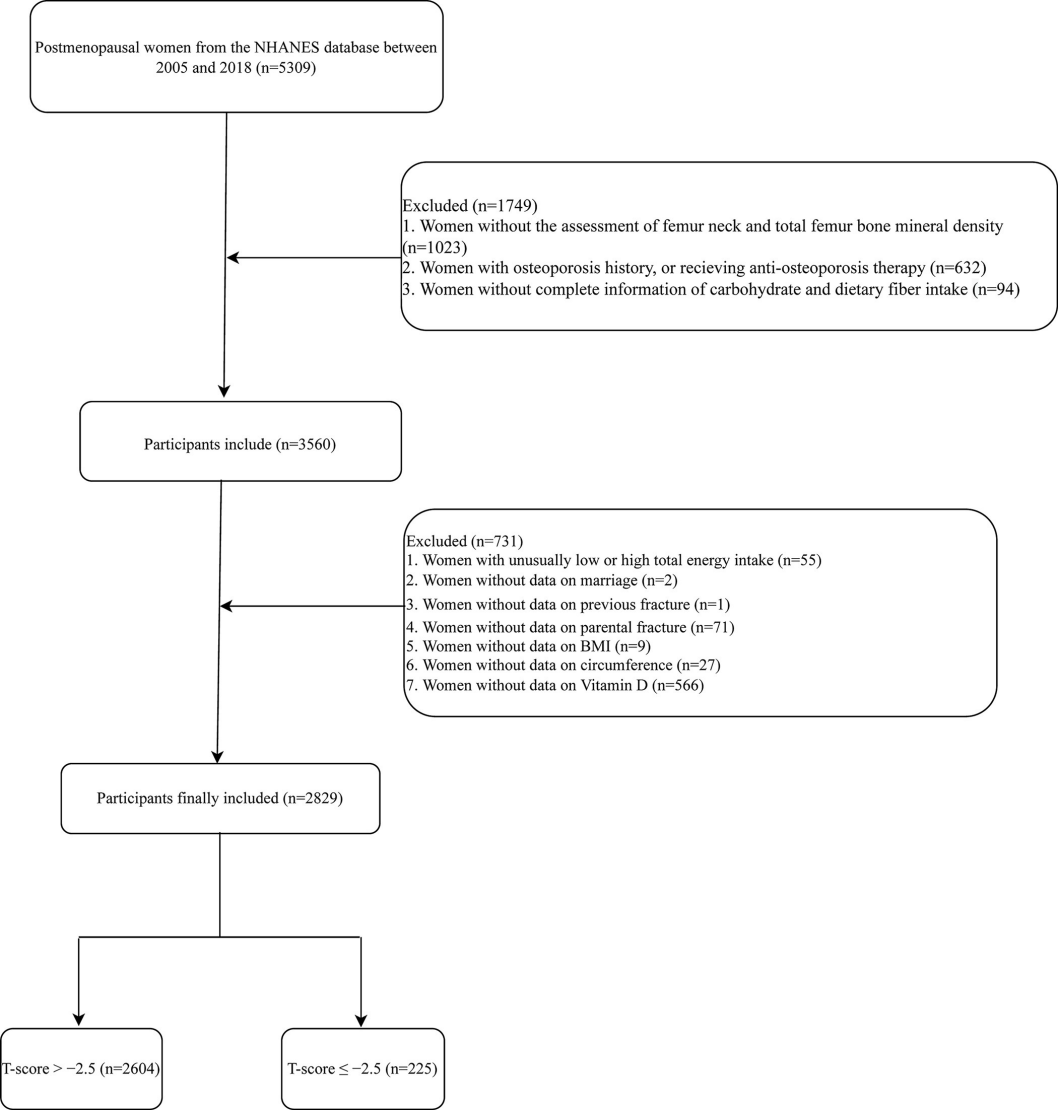
The screen process of the participants. BMI: body mass index; NHANES: National Health and Nutrition Examination Survey.

A total of 5309 postmenopausal women were identified from the NHANES database. Among them, participants without the assessment of femur neck and total femur BMD (n = 1023), participants with osteoporosis history, or received anti-osteoporosis therapy (n = 632), and those without complete information of carbohydrate and dietary fiber intake (n = 94) were excluded. Participants with unusually low or high total energy intake (<500 kcal/day or >5000 kcal/day) (n = 55), and women with no data on marriage (n = 2), previous fracture (n = 1), parental fracture (n = 71), BMI (n = 9), circumference (n = 27) and Vitamin D (n = 566) were excluded. Finally, 2829 postmenopausal women were included. The flow chat of screen process of the participants was exhibited in Fig 1.

Compared with people without osteoporosis, the mean dietary fiber intake in people with osteoporosis was lower than the non-osteoporosis group (14.05 gm vs 15.72 gm). The mean carbohydrate to fiber ratio in the non-osteoporosis group was lower than the osteoporosis group (2.66 vs 2.78). The mean age in the non-osteoporosis group was lower than the osteoporosis group (61.14 years vs 70.77 years). The percentages of participants with physical activity ≥450 MET × min/week in the non-osteoporosis group was higher than the osteoporosis group (57.40% vs 47.61%). The previous fracture rate of the non-osteoporosis group was lower than the osteoporosis group (0.88% vs 5.18%) (Table 1).

**Table 1:** Comparisons of characteristics of postmenopausal women with or without osteoporosis (T score <-2.5).

Variables	Total (n = 2829)	T-score > −2.5 (n = 2604)	T-score ≤ −2.5 (n = 225)	Statistics	*P*
General characteristics					
Age, years, Mean (S.E)	61.77 (0.25)	61.14 (0.23)	70.77 (0.86)	t = -12.07	<0.001
Race, n (%)				χ^2^ = 16.44	0.002
Mexican American	418 (5.53)	400 (5.71)	18 (2.92)		
Other Hispanic	307 (4.51)	281 (4.46)	26 (5.20)		
Non-Hispanic White	1357 (74.67)	1220 (74.37)	137 (79.00)		
Non-Hispanic Black	531 (9.13)	512 (9.50)	19 (3.78)		
Other Race—Including Multi-Racial	216 (6.16)	191 (5.96)	25 (9.11)		
Education, n (%)				χ^2^ = 19.63	<0.001
Less than 9th grade	341 (5.49)	299 (5.03)	42 (12.14)		
9-11th grade (Includes 12th grade with no diploma)	406 (10.60)	368 (10.29)	38 (15.01)		
High school graduate/GED or equivalent	715 (27.26)	657 (27.38)	58 (25.49)		
Some college or AA degree	799 (30.34)	744 (30.11)	55 (33.56)		
College graduate or above	568 (26.32)	536 (27.18)	32 (13.80)		
Marriage, n (%)				χ^2^ = 68.95	<0.001
Married	1409 (57.42)	1327 (58.58)	82 (40.51)		
Widowed	544 (15.14)	460 (13.70)	84 (35.97)		
Divorced	513 (17.77)	480 (17.82)	33 (17.08)		
Separated	92 (1.62)	86 (1.65)	6 (1.23)		
Never married	196 (5.39)	178 (5.47)	18 (4.28)		
Living with partner	75 (2.65)	73 (2.77)	2 (0.94)		
Body mass index, kg/m^2^, Mean (S.E)	28.94 (0.18)	29.23 (0.18)	24.68 (0.35)	t = 11.55	<0.001
Overweight, n (%)				χ^2^ = 54.31	<0.001
No	741 (28.67)	606 (26.82)	135 (55.48)		
Yes	2088 (71.33)	1998 (73.18)	90 (44.52)		
Circumference, cm, Mean (S.E)	97.98 (0.43)	98.54 (0.44)	89.80 (0.85)	t = 9.42	<0.001
Poverty-to-income ratio, n (%)				χ^2^ = 5.03	0.081
<1.0	447 (9.57)	405 (9.29)	42 (13.58)		
≥1.0	2110 (82.71)	1951 (83.15)	159 (76.38)		
Unknown	272 (7.72)	248 (7.56)	24 (10.03)		
Drinking, n (%)				χ^2^ = 1.22	0.269
≤twice/week	2534 (86.35)	2334 (86.58)	200 (83.00)		
>twice/week	295 (13.65)	270 (13.42)	25 (17.00)		
Smoking, n (%)				χ^2^ = 0.06	0.805
No	1715 (58.23)	1578 (58.15)	137 (59.28)		
Yes	1114 (41.77)	1026 (41.85)	88 (40.72)		
Physical activity, n (%)				χ^2^ = 14.27	<0.001
<450 MET ×min/week	354 (12.22)	334 (12.54)	20 (7.66)		
≥450 MET × min/week	1447 (56.77)	1355 (57.40)	92 (47.61)		
Unknown	1028 (31.01)	915 (30.06)	113 (44.73)		
**Complications**					
Hypertension, n (%)				χ^2^ = 6.27	0.012
No	1068 (44.02)	992 (44.65)	76 (34.81)		
Yes	1761 (55.98)	1612 (55.35)	149 (65.19)		
Diabetes, n (%)				χ^2^ = 1.03	0.310
No	2161 (81.47)	1977 (81.26)	184 (84.51)		
Yes	668 (18.53)	627 (18.74)	41 (15.49)		
Dyslipidemia, n (%)				χ^2^ = 3.80	0.051
No	458 (16.45)	410 (15.97)	48 (23.42)		
Yes	2371 (83.55)	2194 (84.03)	177 (76.58)		
Previous fracture, n (%)				χ^2^ = 22.84	<0.001
No	2798 (98.85)	2581 (99.12)	217 (94.82)		
Yes	31 (1.15)	23 (0.88)	8 (5.18)		
Parental fracture, n (%)				χ^2^ = 2.47	0.116
No	2523 (86.90)	2328 (87.31)	195 (80.98)		
Yes	306 (13.10)	276 (12.69)	30 (19.02)		
History of physician-diagnosed osteoporosis, n (%)				χ^2^ = 25.20	<0.001
No	2646 (93.58)	2457 (94.23)	189 (84.28)		
Yes	183 (6.42)	147 (5.77)	36 (15.72)		
**Eating habits**					
Carbohydrate-fiber ratio, Mean (S.E)	2.67 (0.02)	2.66 (0.02)	2.78 (0.05)	t = -2.30	0.025
Carbohydrate-fiber ratio, n (%)				χ^2^ = 6.58	0.037
Carbohydrate-fiber ratio≤11.59	949 (33.27)	881 (33.55)	68 (29.31)		
11.59 <Carbohydrate-fiber ratio≤17.09	916 (33.40)	853 (33.76)	63 (28.22)		
Carbohydrate-fiber ratio>17.09	964 (33.32)	870 (32.69)	94 (42.46)		
Carbohydrate, gm, Mean (S.E)	211.64 (2.79)	211.39 (2.90)	215.23 (9.22)	t = -0.40	0.691
Carbohydrate, gm, n (%)				χ^2^ = 2.62	0.269
Carbohydrate≤168.60	1034 (33.26)	937 (32.90)	97 (38.56)		
168.60<Carbohydrate≤232.19	868 (33.44)	810 (33.90)	58 (26.74)		
Carbohydrate>232.19	927 (33.30)	857 (33.20)	70 (34.70)		
Dietary fiber, gm, Mean (S.E)	15.61 (0.31)	15.72 (0.33)	14.05 (0.60)	t = 2.43	0.018
Dietary fiber, gm, n (%)				χ^2^ = 6.61	0.037
Dietary fiber≤10.93	988 (33.09)	893 (32.40)	95 (43.05)		
10.93 <Dietary fiber≤17.65	959 (33.12)	892 (33.28)	67 (30.78)		
Dietary fiber>17.65	882 (33.80)	819 (34.32)	63 (26.17)		
Cotinine, ng/mL, Mean (S.E)	39.79 (3.48)	38.41 (3.55)	59.65 (9.99)	t = -2.09	0.041
25[OH]D, nmol/L, Mean (S.E)	76.79 (1.03)	76.70 (1.08)	78.08 (2.99)	t = -0.44	0.663
Alkaline-phosphatase, IU/L, Mean (S.E)	75.30 (0.62)	74.82 (0.65)	82.24 (2.36)	t = -2.97	0.004
Calcium values in laboratory, mg/dL, Mean (S.E)	9.48 (0.02)	9.48 (0.02)	9.46 (0.04)	t = 0.54	0.593
Phosphorus, mg/dL, Mean (S.E)	3.91 (0.01)	3.91 (0.01)	3.99 (0.05)	t = -1.46	0.150
Calcium, mg, Mean (S.E)	1172.56 (19.78)	1177.73 (20.46)	1097.68 (64.06)	t = 1.21	0.232
Vitamin D, mcg, Mean (S.E)	19.48 (1.06)	19.41 (1.10)	20.49 (4.22)	t = -0.25	0.805
Protein intake, gm, Mean (S.E)	66.95 (0.76)	67.44 (0.79)	59.84 (2.43)	t = 3.06	0.003
Total energy, kcal, Mean (S.E)	1740.12 (19.77)	1744.21 (20.69)	1680.83 (55.85)	t = 1.08	0.286
**Drug use**					
Estrogens, n (%)				χ^2^ = 10.98	<0.001
No	2675 (91.83)	2454 (91.37)	221 (98.50)		
Yes	154 (8.17)	150 (8.63)	4 (1.50)		
Nx drug, n (%)				χ^2^ = 2.77	0.096
No	2761 (97.62)	2546 (97.73)	215 (96.04)		
Yes	68 (2.38)	58 (2.27)	10 (3.96)		
Kz drug, n (%)				χ^2^ = 0.77	0.380
No	2389 (82.51)	2200 (82.73)	189 (79.42)		
Yes	440 (17.49)	404 (17.27)	36 (20.58)		
Glucocorticoid use, n (%)				χ^2^ = 1.27	0.259
No	2761 (98.04)	2543 (98.13)	218 (96.75)		
Yes	68 (1.96)	61 (1.87)	7 (3.25)		

AA: Associate of Arts; GED: general educational development; MET: metabolic equivalent of task (MET); S.E: standard error; 25[OH]D: 25-hydroxyvitamin

* The detailed race in other race group was not possible to be identified.

### Associations of carbohydrate, dietary fiber, carbohydrate to fiber ratio with the odds ratio of osteoporosis in postmenopausal women

As observed in S3 Table, we found that age, race, education, marriage, poverty-to-income ratio, physical activity, hypertension, previous fracture, BMI, circumference, cotinine, protein intake, history of physician-diagnosed osteoporosis or osteoporosis treatment, and estrogens treatment were confounding factors associated with osteoporosis. In previous study, total energy intake was reported to be associated with muscle mass [20], and low energy intakes might contribute to the declining intakes of calcium and other minerals [21]. Thus, we also adjusted total energy as a covariate.

Higher dietary fiber intake was linked with decreased odds ratio of osteoporosis in the adjusted model (OR = 0.96, 95%CI: 0.93 to 0.99). Compared with women with dietary fiber ≤10.93 gm, those with dietary fiber of 10.93 gm-17.65 gm (OR = 0.57, 95%CI: 0.34 to 0.98) and dietary fiber>17.65 gm (OR = 0.47, 95%CI: 0.23 to 0.99) were related to lowered odds ratio of osteoporosis. The odds ratio of osteoporosis in postmenopausal women was higher as the increase of carbohydrate to fiber ratio (OR = 1.80, 95%CI: 1.10 to 2.96). Carbohydrate to fiber ratio >17.09 was related to increased odds ratio of osteoporosis after adjusting for confounders (OR = 1.63, 95%CI: 1.04 to 2.56) (Table 2).

**Table 2:** Associations of carbohydrate, dietary fiber, or carbohydrate to fiber ratio with the prevalence of osteoporosis (T score <-2.5) in postmenopausal women.

Variables	n (%)	Model 1		Model 2		Model 3	
		OR (95%*CI)*	*P*	OR (95%CI)	*P*	OR (95%CI)	*P*
Carbohydrate (continuous variable)		1.00 (1.00–1.00)	0.686	1.00 (1.00–1.00)	0.145	1.00 (1.00–1.01)	0.728
Carbohydrate tertiles							
≤168.60 gm	1034 (33.26)	Ref		Ref		Ref	
168.60 gm-32.19 gm	868 (33.44)	0.67 (0.40–1.14)	0.138	0.74 (0.42–1.30)	0.288	0.71 (0.36–1.41)	0.317
>232.19 gm	927 (33.30)	0.89 (0.57–1.40)	0.615	1.08 (0.68–1.70)	0.752	0.87 (0.41–1.83)	0.701
Dietary fiber (continuous variable)		0.97 (0.95–0.99)	0.031	0.97 (0.95–0.99)	0.019	0.96 (0.93–0.99)	0.012
Dietary fiber tertiles							
≤10.93 gm	988 (33.09)	Ref		Ref		Ref	
10.93 gm-17.65 gm	959 (33.12)	0.70 (0.46–1.05)	0.086	0.60 (0.39–0.93)	0.023	0.57 (0.34–0.98)	0.041
>17.65 gm	882 (33.80)	0.57 (0.35–0.95)	0.030	0.53 (0.32–0.89)	0.018	0.47 (0.23–0.99)	0.048
Carbohydrate-fiber ratio (continuous variable)		1.62 (1.09–2.41)	0.018	2.44 (1.51–3.94)	<0.001	1.80 (1.10–2.96)	0.021
Carbohydrate-fiber ratio tertiles							
≤11.59	949 (33.27)	Ref		Ref		Ref	
11.59–17.09	916 (33.40)	0.96 (0.64–1.43)	0.826	1.02 (0.68–1.54)	0.907	0.93 (0.58–1.50)	0.758
>17.09	964 (33.32)	1.49 (1.01–2.18)	0.042	1.97 (1.35–2.89)	<0.001	1.63 (1.04–2.56)	0.033

CI: confidence interval; OR: odds ratio; Ref: reference; 25[OH]D: 25-hydroxyvitamin D

Model 1, no variables were adjusted

Model 2, age and race were adjusted

Model 3, age, race, education, marriage, poverty-to-income ratio, physical activity, hypertension, previous fracture, BMI, circumference, cotinine, protein intake, history of physician-diagnosed osteoporosis or osteoporosis treatment, and estrogens treatment, and total energy were adjusted.

### Associations of carbohydrate, dietary fiber, or carbohydrate to fiber ratio with hip BMD in postmenopausal women

According to the data in Table 3, total femur BMD was higher in dietary fiber of 10.93 gm-17.65 gm (β = 0.018, 95%CI: 0.004 to 0.032), and dietary fiber>17.65 gm (β = 0.018, 95%CI: 0.005 to 0.032). Compared to the carbohydrate to fiber ratio ≤11.59 group, carbohydrate to fiber ratio >17.09 was associated with decreased total femur BMD in postmenopausal women (β = -0.015, 95%CI: -0.028 to -0.001). Higher carbohydrate alone was not significantly associated with total femur BMD (*P*>0.05).

**Table 3:** Associations of carbohydrate, dietary fiber, or carbohydrate to fiber ratio with hip BMD in postmenopausal women.

Variables		n (%)	Model 1		Model 2		Model 3	
			β (95%CI*)*	*P*	β (95%CI)	*P*	β (95%CI)	*P*
	Total femur BMD							
Carbohydrate (continuous variable)			<0.001(-0.000, <0.001)	0.913	-0.000 (-0.000, <0.001)	0.638	-0.000 (-0.000, <0.001)	0.728
Carbohydrate tertiles								
≤168.60 gm		1034 (33.26)	Ref		Ref		Ref	
168.60 gm-32.19 gm		868 (33.44)	0.016 (-0.001, 0.034)	0.059	0.015 (-0.002, 0.032)	0.080	0.019 (0.004, 0.033)	0.014
>232.19 gm		927 (33.30)	0.010 (-0.009, 0.029)	0.287	0.004 (-0.014, 0.022)	0.680	0.010 (-0.010, 0.029)	0.335
Dietary fiber (continuous variable)			<0.001(-0.000, 0.001)	0.354	<0.001(-0.000, 0.001)	0.060	<0.001(-0.000, 0.001)	0.065
Dietary fiber tertiles								
≤10.93 gm		988 (33.09)	Ref		Ref		Ref	
10.93 gm-17.65 gm		959 (33.12)	0.013 (-0.005, 0.030)	0.147	0.020 (0.003, 0.037)	0.024	0.018 (0.004, 0.032)	0.010
>17.65 gm		882 (33.80)	0.012 (-0.004, 0.029)	0.149	0.019 (0.003, 0.035)	0.020	0.018 (0.005, 0.032)	0.007
Carbohydrate-fiber ratio (continuous variable)			-0.004 (-0.019, 0.012)	0.634	-0.020 (-0.035, -0.004)	0.016	-0.008 (-0.021, 0.006)	0.255
Carbohydrate-fiber ratio tertiles								
≤11.59		949 (33.27)	Ref		Ref		Ref	
11.59–17.09		916 (33.40)	0.006 (-0.012, 0.025)	0.485	0.002 (-0.016, 0.020)	0.809	0.006 (-0.010, 0.023)	0.447
>17.09		964 (33.32)	-0.005 (-0.023, 0.012)	0.542	-0.020 (-0.037, -0.003)	0.020	-0.015 (-0.028, -0.001)	0.035
	Femur neck BMD							
Carbohydrate (continuous variable)			-0.000 (-0.000, <0.001)	0.704	-0.000 (-0.000, <0.001)	0.294	-0.000 (-0.000, <0.001)	0.431
Carbohydrate tertiles								
≤168.60 gm		1034 (33.26)	Ref		Ref		Ref	
168.60 gm-232.19 gm		868 (33.44)	0.014 (-0.001, 0.030)	0.061	0.014 (-0.001, 0.028)	0.068	0.017 (0.002, 0.032)	0.030
>232.19 gm		927 (33.30)	0.002 (-0.015, 0.019)	0.822	-0.005 (-0.021, 0.011)	0.553	<0.001(-0.020, 0.020)	0.970
Dietary fiber (continuous variable)								
Dietary fiber tertiles			<0.001 (-0.001, <0.001)	0.862	<0.001 (-0.000, 0.001)	0.103	<0.001 (-0.000, 0.001)	0.054
≤10.93 gm		988 (33.09)	Ref		Ref		Ref	
10.93 gm-17.65 gm		959 (33.12)	0.009 (-0.007, 0.025)	0.262	0.018 (0.003, 0.034)	0.021	0.019 (0.006, 0.033)	0.004
>17.65 gm		882 (33.80)	0.007 (-0.008, 0.021)	0.368	0.016 (0.003, 0.030)	0.019	0.022 (0.007, 0.036)	0.004
Carbohydrate-fiber ratio (continuous variable)			-0.003 (-0.017, 0.012)	0.717	-0.023 (-0.037, -0.008)	0.003	-0.015 (-0.028, -0.001)	0.041
Carbohydrate-fiber ratio tertiles								
≤11.59		949 (33.27)	Ref		Ref		Ref	
11.59–17.09		916 (33.40)	-0.001 (-0.018, 0.017)	0.953	-0.006 (-0.023, 0.012)	0.523	-0.003 (-0.019, 0.014)	0.762
>17.09		964 (33.32)	-0.005 (-0.020, 0.011)	0.567	-0.023 (-0.038, -0.009)	0.002	-0.020 (-0.033, -0.006)	0.005

BMD: bone mineral density; CI: confidence interval; Ref: reference; 25[OH]D: 25-hydroxyvitamin D

Model 1 adjusted for no variable

Model 2 adjusted for age and race

Model 3 adjusted for age, race, education, marriage, poverty-to-income ratio, physical activity, hypertension, previous fracture, BMI, circumference, cotinine, protein intake, history of physician-diagnosed osteoporosis or osteoporosis treatment, and estrogens treatment, and total energy.

Higher carbohydrate was not significantly associated with femur neck BMD (*P*>0.05). Dietary fiber intake of 10.93 gm-17.65 gm (β = 0.019, 95%CI: 0.006 to 0.036), and dietary fiber>17.65 gm (β = 0.022, 95%CI: 0.007,0.036) were correlated with elevated femur neck BMD in postmenopausal women. The femur neck BMD in postmenopausal women was decreased with the increase of carbohydrate to fiber ratio (β = -0.015, 95%CI: -0.028 to -0.001). Compared to people with carbohydrate to fiber ratio ≤11.59, the femur neck BMD was decreased in postmenopausal women with carbohydrate to fiber ratio >17.09 (β = -0.020, 95%CI: -0.033 to -0.006) (Table 3).

### Subgroup analysis of associations of carbohydrate, dietary fiber, carbohydrate to fiber ratio with the odds ratio of osteoporosis, and with total femur BMD or femur neck BMD

In non-overweight postmenopausal women, dietary intake was identified to be a protective factor for osteoporosis with an OR value of (OR = 0.95, 95%CI: 0.91 to 0.99). Dietary fiber >17.65 gm was correlated with lowered odds ratio of osteoporosis in non-overweight postmenopausal women (OR = 0.33, 95%CI: 0.13 to 0.87). Increased carbohydrate to fiber ratio was related to higher odds ratio of osteoporosis (OR = 1.99, 95%CI: 1.12 to 3.53). Carbohydrate to fiber ratio >17.09 was correlated with increased odds of osteoporosis (OR = 2.12, 95%CI: 1.08 to 4.16) (Table 4).

**Table 4:** Subgroup analysis of associations of carbohydrate, dietary fiber or carbohydrate to fiber ratio with osteoporosis (T score <-2.5), and with hip BMD, respectively.

Variables	Osteoporosis			Total femur BMD			femur neck BMD		
	OR (95%CI*)*	*P*	*P* for interaction	β (95%CI)	*P*	*P for interaction*	β (95%CI)	*P*	*P* for interaction
Body mass index≥25kg/m^2^ (n = 2088)			0.087			0.149			0.396
Carbohydrate (continuous variable)	1.00 (0.99–1.01)	0.513		<0.001 (-0.000, <0.001)	0.472		<0.001 (-0.000, <0.001)	0.616	
Carbohydrate tertiles									
≤168.60 gm	Ref			Ref			Ref		
168.60 gm-232.19 gm	1.16 (0.45–2.96)	0.754		0.014 (-0.004, 0.032)	0.114		0.016 (-0.001,0.033)	0.073	
>232.19 gm	0.61 (0.22–1.71)	0.344		0.015 (-0.009, 0.039)	0.213		0.007 (-0.017,0.031)	0.545	
Body mass index≥25kg/m^2^ (n = 2088)			0.468			0.109			0.055
Dietary fiber (continuous variable)	0.97 (0.94–1.01)	0.196		<0.001 (<0.001, 0.002)	0.041		0.001 (-0.000,0.002)	0.054	
Dietary fiber tertiles									
≤10.93 gm	Ref			Ref			Ref		
10.93 gm -17.65 gm	0.75 (0.38–1.48)	0.402		0.018 (0.003, 0.034)	0.021		0.020 (0.006,0.033)	0.006	
>17.65 gm	0.79 (0.33–1.87)	0.585		0.023 (0.007, 0.038)	0.004		0.026 (0.009,0.042)	0.003	
Body mass index ≥25kg/m2 (n = 2088)			0.089			0.392			0.230
Carbohydrate-fiber ratio (continuous variable)	1.38 (0.61–3.12)	0.429		-0.011 (-0.028, 0.006)	0.188		-0.016 (-0.035,0.002)	0.084	
Carbohydrate-fiber ratio tertiles									
≤11.59	Ref			Ref			Ref		
11.59–17.09	0.89 (0.43–1.81)	0.735		0.002 (-0.020, 0.024)	0.846		-0.006 (-0.026,0.013)	0.515	
>17.09	1.09 (0.61–1.96)	0.763		-0.017 (-0.034, <0.001)	0.058		-0.020 (-0.037, -0.003)	0.022	
Body mass index <25kg/m^2^ (n = 741)									
Carbohydrate (continuous variable)	1.00 (1.00–1.01)	0.618		-0.000 (-0.000, <0.001)	0.349		-0.000 (-0.000, <0.001)	0.158	
Carbohydrate tertiles									
≤168.60 gm	Ref			Ref			Ref		
168.60 gm-232.19 gm	0.49 (0.22–1.10)	0.084		0.032 (0.011, 0.054)	0.004		0.022 (-0.002,0.046)	0.075	
>232.19 gm	1.10 (0.40–3.04)	0.856		0.003 (-0.029, 0.036)	0.830		-0.012 (-0.048,0.023)	0.496	
Body mass index <25kg/m^2^ (n = 741)									
Dietary fiber (continuous variable)	0.95 (0.91–0.99)	0.021		<0.001 (-0.001, 0.002)	0.953		<0.001 (-0.001, 0.002)	0.727	
Dietary fiber tertiles									
≤10.93 gm	Ref			Ref			Ref		
10.93 gm-17.65 gm	0.58 (0.30–1.10)	0.095		0.017 (-0.008, 0.042)	0.172		0.022 (-0.004,0.047)	0.095	
>17.65 gm	0.33 (0.13–0.87)	0.025		0.008 (-0.020, 0.037)	0.565		0.013 (-0.018,0.045)	0.397	
Body mass index <25kg/m^2^ (n = 741)									
Carbohydrate-fiber ratio (continuous variable)	1.99 (1.12–3.53)	0.019		0.002 (-0.020, 0.024)	0.852		-0.009 (-0.030,0.012)	0.375	
Carbohydrate-fiber ratio tertiles									
≤11.59	Ref			Ref			Ref		
11.59–17.09	1.09 (0.61–1.92)	0.775		0.014 (-0.011, 0.039)	0.276		0.004 (-0.024, 0.033)	0.763	
>17.09	2.12 (1.08–4.16)	0.030		-0.008 (-0.037, 0.021)	0.568		-0.016 (-0.046, 0.013)	0.276	

OR: odds ratio; CI: confidence interval; 25[OH]D: 25-hydroxyvitamin D; Ref: Reference; BMD: bone mineral density

Adjusted for age, race, education, marriage, poverty-to-income ratio, physical activity, hypertension, previous fracture, circumference, cotinine, protein intake, history of physician-diagnosed osteoporosis or osteoporosis treatment, and estrogens treatment, and total energy.

In non-overweight postmenopausal women, total femur BMD was increased in carbohydrate intake of 168.60 gm-232.19 gm group relative to carbohydrate ≤168.60 gm group (β = 0.032, 95%CI: 0.011 to 0.054). Dietary fiber intake was associated with increased total femur BMD (β<0.001, 95%CI: <0.001–0.002). Compared to dietary fiber ≤ Q_1_ group, dietary fiber intake of 10.93 gm-17.65 gm (β = 0.018, 95%CI: 0.003 to 0.034) and dietary fiber intake>17.65 gm (β = 0.023, 95%CI: 0.007 to 0.038) were associated with increased total femur BMD in overweight women (Table 4).

In non-overweight women, dietary fiber intake ≤10.93 gm, BMD at femur neck was increased in those with dietary fiber intake of 10.93 gm-17.65 gm (β = 0.020, 95%CI: 0.006 to 0.033) and dietary fiber intake>17.65 gm (β = 0.026, 95%CI: 0.009 to 0.042) in overweight postmenopausal women. Carbohydrate to fiber ratio >17.09 (β = -0.020, 95%CI: -0.037 to -0.003) were associated with decreased BMD at femur neck in overweight postmenopausal women. (Table 4). In addition, no interaction effect between BMI and carbohydrate, dietary fiber, or carbohydrate to fiber ratio was identified on the odds ratio of osteoporosis in postmenopausal women (*P*>0.05).

## Discussion

The present study analyzed the associations of carbohydrate to dietary fiber ratio with osteoporosis and BMD in postmenopausal women. The results demonstrated that dietary fiber intake >10.93 gm was correlated with decreased odds of osteoporosis. Carbohydrate to fiber ratio >17.09 was associated with increased prevalence of osteoporosis and decreased hip BMD in postmenopausal women.

Carbohydrates are essential for supporting bodily functions and physical activity through providing the body with glucose [22]. The abusive consumption of refined, simple, and low-quality carbohydrates has become a crucial factor in the development of many diseases [23], including bone disease. Previously, Gao et al. observed that higher percentage of energy intake from carbohydrate was associated with lower T-score and higher risk of low BMD [8]. The consumption of diets high in carbohydrates, particularly monosaccharides (such as glucose) and disaccharides (such as sucrose), appears to have a detrimental effect on BMD [24]. In the current study, carbohydrate intake of 168.60 gm-232.19 gm might increase the hip BMD in women, indicating that too high or too low carbohydrate intake might not good for bone health in women. Too low carbohydrate intake might correlate with trends of diseases such as insulin resistance and metabolic acidosis in healthy lean individuals [25]. Dietary fiber is a class of carbohydrate polymers, which is associated with the risk and prognosis of multiple chronic diseases [26, 27]. In a previous study, the researchers found that higher total dietary fiber intake had a moderate effect on reducing hip bone loss in males, while vegetable fiber exhibited significant protective effects against spinal bone loss in females [28]. Several studies have demonstrated a positive correlation between increased consumption of fruits and vegetables and higher bone mineral density, as well as a reduced risk of bone loss and fractures [29, 30]. Zhou et al. found that a higher intake of dietary fiber was positively associated with heel BMD in individuals aged 40–69 years, regardless of gender [11]. These findings gave support to the results in our study. We found that higher dietary fiber intake was correlated with decreased risk of osteoporosis and elevated femur neck BMD in postmenopausal women.

Additionally, higher carbohydrate to fiber ratio was identified to be associated with elevated odds ratio of osteoporosis, lower hip BMD in postmenopausal women. Previous evidence suggested that carbohydrate to fiber ratio were correlated with an increased risk of metabolic disorders [31], type 2 diabetes in US women [32], and coronary heart disease [33]. The potential mechanism might be that high carbohydrates can elevate glucose levels, which negatively impact osteoblast and osteoclast function, and this includes alteration of the insulin signaling pathway and inhibition of osteoblast cells, leading to abnormal bone metabolism and an increased risk of fractures [34, 35]. Higher glucose levels can lead to oxidative stress and inflammation, which in turn decrease osteoblast activity and increase bone resorption by inducing acidosis, ultimately negatively impacting bone health [36]. On the other hand, dietary fiber is sourced from whole-grain cereals, fruits, vegetables, and legumes, which containing essential nutritional elements such as calcium and vitamin D that are crucial in maintaining bone mass [37, 38]. The carbohydrate to fiber ratio might offer a more comprehensive assessment of an individual’s diet compared to separate measurements of carbohydrate or fiber intake [39], which suggested that postmenopausal women might be necessary to be careful of their carbohydrate and fiber intake during diet, especially the dietary quality of postmenopausal women. Correct nutrition consists of the ingestion of macronutrients including proteins, lipids, and carbohydrates, as well as micronutrients in food and in water [40]. To keep the homeostasis of bone health, appropriate ratio of carbohydrate and fiber intake have biological plausibility for the management of bone health.

This study evaluated the associations of carbohydrate to dietary fiber ratio with osteoporosis and BMD at different sites in postmenopausal women based on large and representative sample size from the NHANES database. The findings might provide a reference for the dietary guidance of postmenopausal women. Several limitations were found in our study. Firstly, the NHANES data were cross-sectional, and causal associations between carbohydrate to fiber ratio with osteoporosis and BMD could not be identified in this cross-sectional study. Secondly, only data from total femur and femur neck BMDs were recorded in the NHANES database, data on other sites such as the lumbar spine BMD were not complete, which might potentially underestimate the prevalence of osteoporosis in postmenopausal women. Thirdly, although some confounding factor associated with the odds ratio of osteoporosis such as estrogens treatment, were adjusted, there might be residual or unmeasured confounding not included, which might affect the interpretation of the results.

## Conclusions

The current study evaluated the associations of carbohydrate to dietary fiber ratio with osteoporosis and BMD in postmenopausal women, which found that a high carbohydrate/fiber ratio >17.09 is associated with an increased risk of osteoporosis and lower hip BMD and high fiber intake is associated with less osteoporosis and higher hip BMD in postmenopausal women.

## Supporting information

S1 Table The number and percentage of missing values. (DOCX)

S2 Table Sensitivity analysis of the data before and after manipulation of the missing values. (DOCX)

S3 Table The weighted univariable logistic regression screening potential covariates. (DOCX)
