# Supplementary material for: Association of dietary carbohydrate and fiber ratio with postmenopausal bone mineral density and prevalence of osteoporosis: A cross-sectional study
Source: PLoS One. 2024 Feb 14;19(2):e0297332. doi: 10.1371/journal.pone.0297332 (PMC10866481; doi:10.1371/journal.pone.0297332)
Supplement: S1 Table — (DOCX) [file pone.0297332.s001.docx]

S1 Table The number and percentage of missing values

| Variables | n (%) |
| --- | --- |
| Education | 2 (0.07%) |
| Poverty-to-income ratio | 272 (9.61%) |
| Drinking | 82 (2.9%) |
| Physical activity | 1028 (36.34%) |
| Cotinine | 96 (3.39%) |
| 25[OH]D | 180 (6.36%) |
| Alkaline-phosphatase | 111 (3.92%) |
| Calcium | 111 (3.92%) |
| Phosphorus | 112 (3.96%) |

25[OH]D: 25-hydroxyvitamin D
