# Supplementary material for: Association of dietary carbohydrate and fiber ratio with postmenopausal bone mineral density and prevalence of osteoporosis: A cross-sectional study
Source: PLoS One. 2024 Feb 14;19(2):e0297332. doi: 10.1371/journal.pone.0297332 (PMC10866481; doi:10.1371/journal.pone.0297332)
Supplement: S2 Table — (DOCX) [file pone.0297332.s002.docx]

S2 Table Sensitivity analysis of the data before and after manipulation of the missing values

| Variables | Before manipulation | After manipulation | Statistics | *P* |
| --- | --- | --- | --- | --- |
| Education, n (%) |  |  | χ^2^=7.77 | 0.100 |
| Less than 9th grade | 339 (5.48) | 341 (5.49) |  |  |
| 9-11th grade (Includes 12th grade with no diploma) | 406 (10.60) | 406 (10.60) |  |  |
| High school graduate/GED or equivalent | 715 (27.26) | 715 (27.26) |  |  |
| Some college or AA degree | 799 (30.34) | 799 (30.34) |  |  |
| College graduate or above | 568 (26.32) | 568 (26.32) |  |  |
| Drinking, n(%) |  |  | χ^2^=1.44 | 0.229 |
| ≤twice/week | 2457 (86.22) | 2534 (86.35) |  |  |
| >twice/week | 290 (13.78) | 295 (13.65) |  |  |
| Cotinine, ng/mL, Mean (S.E) | 39.85 (3.59) | 39.79 (3.48) | t=0.20 | 0.844 |
| 25[OH]D, nmol/L, Mean (S.E) | 77.03 (1.05) | 76.79 (1.03) | t=1.47 | 0.146 |
| Alkaline-phosphatase, IU/L, Mean (S.E) | 75.28 (0.61) | 75.30 (0.62) | t=-0.24 | 0.811 |
| Calcium values in laboratory, mg/dL, Mean (S.E) | 9.48 (0.02) | 9.48 (0.02) | t=0.69 | 0.496 |
| Phosphorus, mg/dL, Mean (S.E) | 3.91 (0.01) | 3.91 (0.01) | t=0.24 | 0.810 |

AA: Associate of Arts; GED: General educational development; S.E: Standard error; 25[OH]D: 25-hydroxyvitamin D
