# Supplementary material for: Association of dietary carbohydrate and fiber ratio with postmenopausal bone mineral density and prevalence of osteoporosis: A cross-sectional study
Source: PLoS One. 2024 Feb 14;19(2):e0297332. doi: 10.1371/journal.pone.0297332 (PMC10866481; doi:10.1371/journal.pone.0297332)
Supplement: S3 Table — (DOCX) [file pone.0297332.s003.docx]

S3 Table The weighted univariable logistic regression screening potential covariates

| Variables | OR (95%CI) | *P* |
| --- | --- | --- |
| Age | 1.11 (1.08-1.13) | <0.001 |
| Race |  |  |
| Mexican American | Ref |  |
| Other Hispanic | 2.28 (1.01-5.14) | 0.047 |
| Non-Hispanic White | 2.08 (1.14-3.79) | 0.018 |
| Non-Hispanic Black | 0.78 (0.33-1.87) | 0.571 |
| Other Race - Including Multi-Racial | 2.99 (1.29-6.93) | 0.011 |
| Education |  |  |
| Less than 9th grade | Ref |  |
| 9-11th grade (Includes 12th grade with no diploma) | 0.60 (0.34-1.09) | 0.092 |
| High school graduate/GED or equivalent | 0.39 (0.22-0.67) | <0.001 |
| Some college or AA degree | 0.46 (0.25-0.85) | 0.014 |
| College graduate or above | 0.21 (0.11-0.42) | <0.001 |
| Marriage |  |  |
| Married | Ref |  |
| Widowed | 3.80 (2.46-5.84) | <0.001 |
| Divorced | 1.39 (0.75-2.55) | 0.289 |
| Separated | 1.08 (0.41-2.80) | 0.878 |
| Never married | 1.13 (0.68-1.90) | 0.633 |
| Living with partner | 0.49 (0.10-2.37) | 0.371 |
| Poverty-to-income ratio |  |  |
| <1.0 | Ref |  |
| ≥1.0 | 0.63 (0.41-0.96) | 0.032 |
| Unknown | 0.91 (0.43-1.91) | 0.795 |
| Drinking, n (%) |  |  |
| ≤twice/week | Ref |  |
| >twice/week | 1.32 (0.80-2.18) | 0.270 |
| Smoking |  |  |
| No | Ref |  |
| Yes | 0.95 (0.65-1.39) | 0.806 |
| Physical activity |  |  |
| ＜450 MET × min/week | Ref |  |
| ≥450 MET × min/week | 1.36 (0.66-2.80) | 0.400 |
| Unknown | 2.44 (1.32-4.50) | 0.005 |
| Hypertension |  |  |
| No | Ref |  |
| Yes | 1.51 (1.08-2.11) | 0.016 |
| Diabetes |  |  |
| No | Ref |  |
| Yes | 0.80 (0.51-1.25) | 0.313 |
| Dyslipidemia |  |  |
| No | Ref |  |
| Yes | 0.62 (0.38-1.02) | 0.060 |
| Previous fracture |  |  |
| No | Ref |  |
| Yes | 6.18 (2.55-15.00) | <0.001 |
| Parental fracture |  |  |
| No | Ref |  |
| Yes | 1.62 (0.87-3.00) | 0.125 |
| Glucocorticoid use |  |  |
| No | Ref |  |
| Yes | 1.76 (0.64-4.89) | 0.270 |
| Body mass index | 0.85 (0.82-0.89) | <0.001 |
| Circumference | 0.95 (0.94-0.97) | <0.001 |
| Cotinine | 1.01 (1.01-1.01) | 0.017 |
| 25[OH]D | 1.00 (1.00-1.01) | 0.656 |
| Alkaline-phosphatase | 1.01 (1.01-1.02) | <0.001 |
| Calcium | 0.88 (0.55-1.42) | 0.599 |
| Phosphorus | 1.35 (0.91-2.01) | 0.137 |
| Calcium | 1.00 (1.00-1.00) | 0.260 |
| Vitamin D | 1.00 (1.00-1.00) | 0.774 |
| Protein intake | 0.99 (0.98-0.99) | 0.009 |
| Osq060 |  |  |
| No | Ref |  |
| Yes | 3.04 (1.88-4.91) | <0.001 |
| Estrogens |  |  |
| No | Ref |  |
| Yes | 0.16 (0.05-0.57) | 0.005 |
| Nx drug |  |  |
| No | Ref |  |
| Yes | 1.78 (0.89-3.56) | 0.104 |
| Kz drug |  |  |
| No | Ref |  |
| Yes | 1.24 (0.76-2.04) | 0.385 |
| Total energy | 1.00 (1.00-1.00) | 0.302 |

AA: Associate of Arts; GED: general educational development; MET: metabolic equivalent of task (MET); S.E: standard error; 25[OH]D: 25-hydroxyvitamin

* The detailed race in other race group was not possible to be identified.
